# miRNA-3473b contributes to neuroinflammation following cerebral ischemia

**DOI:** 10.1038/s41419-017-0014-7

**Published:** 2018-01-09

**Authors:** Xiaoyu Wang, Shuangshuang Chen, Jingshu Ni, Jian Cheng, Jia Jia, Xuechu Zhen

**Affiliations:** 1 Jiangsu Key Laboratory of Neuropsychiatric Diseases Research and College of Pharmaceutical Sciences, Soochow University, Suzhou, Jiangsu 215021 China; 2 College of Pharmaceutical Sciences and the Collaborative Innovation Center for Brain Science, Soochow University, Suzhou, China; 3 Department of Pharmacy, Suzhou Municipal Hospital, Suzhou, China; 4 Jiangsu Key Laboratory of Neuropsychiatric Diseases Research and Institute of Neuroscience, Soochow University, Suzhou, China

## Abstract

MicroRNAs play an essential role in stroke pathology. Here, we investigated the role of a newly identified microRNA, miR-3473b, in stroke pathology. The expression of miR-3473b was upregulated in the cortex and striatum in mice following transient middle cerebral artery occlusion (MCAO). Intracerebroventricular injection of the miR-3473b antagomir prior to MCAO remarkably attenuated ischemia-induced expression of miR-3473b and pro-inflammatory factors in the ischemic brain and decreased infarct volumes in mice following MCAO. Using in vitro approaches, we showed that the miR-3473b antagomir reduced the mRNA and protein levels of pro-inflammatory factors (iNOS, COX-2, TNF-α, and IL-6) in BV2 microglial cells subjected to LPS stimulation. The miR-3473b antagomir also decreased the expression of pro-inflammatory factors in BV2 cells activated with conditioned medium collected from oxygen-glucose deprivation (OGD)-treated neurons. Suppressor of cytokine signaling 3 (SOCS3), a physiological regulator of innate and adaptive immunity, was predicted to be a potential target of miR-3473b. We verified that the miR-3473b mimic decreased SOCS3 expression in BV2 cells. Meanwhile, the miR-3473b antagomir significantly increased both SOCS3 mRNA and protein levels in the BV2 cells treated with LPS as well as in the ischemic brain. By using the dual luciferase assay, we further showed that the 3′-untranslational region of SOCS3 was directly targeted by miR-3473b. In conclusion, induction of miR-3473b, which is likely targeted to SOCS3, contributes to stroke pathogenesis by enhancing post-stroke neuroinflammation injury.

## Introduction

Ischemic stroke represents a major public health problem. To develop effective therapies, sustained effort has been devoted to understanding the mechanisms of ischemic cerebral injury. The inflammation and immune responses contribute to tissue damage and repair, which plays a pivotal role in stroke pathogenesis^1^. Thus, targeting stroke-induced neuroinflammation is emerging as an attractive strategy for stroke treatment^2–4^.

MicroRNAs (miRNAs) are endogenous, short (~20 nucleotides) single-stranded RNAs. Generally, miRNAs regulate gene expression at a post-transcriptional level via imperfect pairing with the 3′-untranslated regions (3′-UTRs) of target mRNAs. Therefore, miRNAs modulate diverse biological processes, including cell differentiation, the cell cycle, proliferation, apoptosis and the cellular stress response^5^. Growing literature suggests that miRNAs regulate the intracellular pathways of numerous inflammatory mediators^6,7^. Although emerging evidence indicates that miRNAs are altered following both human and rodent stroke^8–10^, information regarding the role of miRNAs in post-stroke inflammatory response regulation and its functional implication remain limited^10^. Recently, we used a deep sequencing approach to examine changes in the miRNA profile of glial cells following MACO in GFAP transgenic mice. We found that miR-3473b was upregulated in the brain following transient cerebral ischemia (data not shown). The biological function of this newly identified miRNA is largely unknown. Interestingly, a recent study suggested that miR-3473b may suppress peripheral macrophage-mediated inflammation^11^. The present study was designed to investigate whether miR-3473b contributes to stroke pathogenesis by modulating microglia-mediated neuroinflammation following cerebral ischemia. The results suggest that post-ischemic induction of miR-3473b contributes to post-ischemic neuroinflammation and exacerbates cerebral ischemic injury by possibly targeting microglial suppressor of cytokine signaling 3 (SOCS3). Inhibiting post-ischemic induction of miR-3473b may represent a novel therapeutic target for ischemic stroke.

## Materials and methods

### Mouse model of transient focal cerebral ischemia

All of the animal experiments were approved by the animal welfare committee of Soochow University and followed the guidance of the NIH for the Care and Use of Laboratory Animals. Male CD-1 mice, weighing 25 to 30 g, were purchased from SLAC Laboratory Animals (Shanghai, China). The mice were anesthetized with isoflurane. Focal cerebral ischemia was produced by 1 h of middle cerebral artery occlusion (MCAO) followed by reperfusion via an intraluminal suture technique, as described previously^10,12^. Briefly, the right common carotid, external and internal carotid arteries were exposed for insertion of a silicon-coated nylon monofilament with a heat-blunted tip (diameter 0.22 ± 0.02 mm). The tip of the filament was advanced to reach the origin of the middle cerebral artery, as indicated by an abrupt reduction in cortical perfusion measured by laser Doppler flowmetry (<30% of the baseline). The surgical site was sutured after the operation was completed. The filament was withdrawn to allow for reperfusion at 1 h post-occlusion. Sham-operated mice underwent the same surgery except for the suture insertion. Rectal temperatures were monitored continuously and maintained at 37 ± 0.5 ˚C with a heating pad.

### MicroRNA sequencing

Transgenic mice with specific expression of eGFP in the glial cells were subjected to 1 h MCAO followed by 6 h of reperfusion. Then, the brain tissues were digested with collagenase (Sigma, St. Louis, USA) and DNase I (Roche, Mannheim, Germany) into single cells and FACS were performed to purify the eGFP positive glial cells from the ischemic cortex and striatum. Total RNA were extracted using the Trizol regent and used for microRNA sequencing analysis (Kangchen, China).

### Behavioral tests

Modified neurological severity scores (mNSS) and corner test were assessed blindly to evaluate neurological deficits as previously reported^10^. The mice were trained for 2 days after receiving intracerebroventricular injection of miR-3473b antagomir or NC antagomir. We performed basal behavioral tests at 1 day before MCAO and the tests were continually performed for 4 days after MCAO. mNSS were composited by sensory, motor, balance and reflex tests and graded on a scale of 0–18. The higher scores, the more severe impairment^13^.

In the corner test, the mice were placed between two pieces of cardboard with an angel of 30. When the mice were forced into the corner, the vibrissae on the both sides were stimulated. As a result, the mice reared and turned back to the open end. The trials were performed 10 times each day. Whereas non-ischemic mice turned without side preference, mice suffering from MCAO preferentially left the corner towards the non-impaired (right) side^10,14^.

### Isolation of RNA and quantitative real-time PCR

RT-PCR was performed to measure the miR-3473b levels in brain tissues following MCAO. Leukocytes were extracted from whole blood after MCAO with ACK lysing buffer (Gibco). We used the mirVana™ miRNA Isolation Kit (Ambion, CA, USA) to isolate the total miRNA. Reverse transcription was performed using a TaqMan MicroRNA reverse transcription kit (Ambion, CA, USA). The levels of mature miR-3473b were determined using a stem-loop real-time PCR system with TaqMan Universal Master Mix II (Ambion, CA, USA). The miR-3473b levels were normalized to those of U6 snRNA. To analyze the mRNA levels of iNOS, TNF-α, and COX-2 in cortical tissue, samples were harvested from the cortex at 24 h post-MCAO. To measure the mRNA levels of iNOS, TNF-α, and COX-2 in BV2 microglial cells, cells were collected 24 h after LPS stimulation or treatment with conditioned medium collected from oxygen-glucose deprivation (OGD) neurons as described^15^. Total RNAs were isolated with RNAiso Plus (Takara, Dalian, China). Total RNA (1 µg) was reverse-transcribed into cDNA using oligo (dT), according to the manufacturer’s instructions (Takara, Dalian, China). Quantitative PCR was performed using the following specific primers:

iNOS, forward primer: 5′-CAGGAGGAGAGAGATCCGATTTA-3′, reverse primer: 5‵-GCATTAGCATGGAAGCAAAGA-3‵;

TNF-α, forward primer: 5′-CATCTTCTCAAAATTCGAGTGACAA-3′, reverse primer: 5‵-TGGGAGTAGACAAGGTACAACCC-3‵;

COX-2, forward primer: 5′-CAGGCTGAACTTCGAAACA-3′, reverse primer: 5‵-GCTCACGAGGCCACTGATACCTA-3‵;

SOCS3, forward primer: 5′-ATGGTCACCCACAGCAAGTTT-3′, reverse primer: 5‵-TCCAGTAGAATCCGCTCTCCT-3‵

GAPDH, forward primer: 5′-TGTGTCCGTCGTGGATCTGA-3′, reverse primer: 5‵-TTGCTGTTGAAGTCGCAGGAG-3‵.

Quantitative real-time PCR was performed using SYBR Premix Ex Taq (TaKaRa, China). The 2^-ΔΔCT^ formula was used for calculations of the relative quantification. The target gene expressions were normalized to GAPDH and expressed as the fold change relative to the expression level of the target gene in the control group.

### Intracerebroventricular injection of the miR-3473b antagomir

The miR-3473b antagomir (CCCGACCUCUCUACCGAGUC) and NC antagomir were purchased from RiboBio (Guangzhou, China). The NC and miR-3473b antagomirs (2.5 µg/2.5 µl) were diluted with 1.25 µl of Entranster^TM^ in vivo transfection reagent (Engreen, Beijing, China). The solution was mixed gently, kept at room temperature for 15 min and then injected intracerebroventricularly (i.c.v.) using a microsyringe (Hamilton, Nevada, USA) under the guidance of a stereotaxic instrument (RWD Life Science). Intracerebroventricular injection was performed according to a previously described method^16^. The stereotaxic coordinates were 0.5 mm posterior, 1.0 mm lateral to the bregma and 2.5–3.0 mm ventral to the bregma.

### Preparation of conditioned medium from OGD-treated neurons

Mouse primary cortical neurons were cultured in complete neurobasal medium supplemented with B27 as previously described^10,17^. To initiate OGD, the culture medium was replaced with OGD buffer (NaCl: 4.09 g; KCl: 186.5 mg; CaCl_2_: 111 mg; HEPES: 2.38 g; Glycine: 1.125 g. Dissolved in 500 ml of distilled water). Then, the neurons were transferred into an incubation chamber flushed with a gas mixture of 95% N_2_ and 5% CO_2_. The cells were kept in the chamber at 37 ˚C for 2.5 h. Following OGD, the cells were fed with complete neurobasal medium and returned to the normoxic condition (5% CO_2_ atmosphere) to allow for re-oxygenation. The supernatants (OGD conditioned medium, OGD CM), were collected 24 h after reperfusion. The supernatants from neuronal cultures without OGD treatment were collected as the controls.

### Cell culture and transfection

The murine BV2 microglial cells were cultured in high-glucose DMEM supplemented with 10% (v/v) heat-inactivated fetal bovine serum, 100 U/mL penicillin, and 100 mg/mL streptomycin. The cells were cultured at 37 °C in a 5% CO_2_ atmosphere. The cells were transfected with 200 nM aliquots of either the miR-3473b antagomir or control antagomir using Lipofectamine® RNAiMAX (ThermoFisher, CA, USA) per the manufacturer’s protocol.

### Enzyme-linked immunosorbent assay (ELISA)

Enzyme-linked immunosorbent assay (ELISA) was used to assess the TNF-α and IL-6 levels in the ischemic brains and supernatants collected from cultured BV2 cells. Ipsilateral and contralateral cortices were harvested from mice subjected to MCAO or sham-operated mice 24 h after reperfusion. The cortical samples were homogenized by ultrasonic wave in radioimmunoprecipitation assay buffer supplemented with a protease inhibitor mixture (Roche, Mannheim, Germany). After the homogenates were centrifuged at 12,000 rpm for 20 min at 4 ˚C, the supernatants were collected. Medium was collected from the BV2 microglial cells 24 h after LPS stimulation or after incubation with the conditioned medium collected from the OGD neurons. A BCA protein assay kit (Tiangen, Beijing, China) was used to measure the protein concentration of the supernatant collected from either cortical tissue or the medium from cultured BV2 cells. TNF-α and IL-6 were measured with commercial ELISA kits (Boster Biosciences Co., Wuhan, China) per the manufacturer’s instructions. A microplate reader (Infinite M200 PRO, Tecan, Switzerland) was used to assess the OD value.

### Dual luciferase target validation and reporter assay

The fragment of the mouse SOCS3 3′-UTR (1484 nt) containing two potential miR-3473b target sites (nucleotides 76–82 and 1301–1307 of the SOCS3 3′-UTR, CCGACCU) and a fragment containing a mutated miR-3473b target site (CCCAGCA) within the seed region were inserted into the luciferase reporter plasmid (pmirGLO, GenePharma). HEK-293 cells were seeded into 24-well plates and co-transfected with 200 ng of the luciferase reporter plasmid and 50 nM of the miR-3473b mimic (GGGCUGGAGAGAUGGCUCAG) using Lipofectamine 2000 (Invitrogen). After 48 h, the firefly and Renilla luciferase activity were measured with the dual-luciferase reporter assay system (Promega) according to the manufacturer’s instructions. Firefly luciferase activity was normalized to Renilla luciferase activity for each transfected well. The experiments were performed in triplicate.

### Western blot analysis

Immunoblotting was performed to assess either the protein level of SOCS3 in HEK cells transfected with the miR-3473b mimic or the protein levels of the pro-inflammatory factors in BV2 microglial cells. To assess the SOCS3 protein level, HEK cells were washed with PBS three times and then lysed in lysis buffer, 48 h after transfection with the miR-3473b mimic (GGGCUGGAGAGAUGGCUCAG). To measure the protein levels of the pro-inflammatory factors, BV2 microglial cells were collected and lysed at either 24 h after LPS stimulation or 24 h after treatment with conditioned medium collected from the OGD neurons. The protein concentrations were determined with a commercial kit (Bio-Rad, Hercules, CA). Sixty micrograms of protein from each sample was separated by SDS-polyacrylamide gel electrophoresis (10%) and then transferred onto nitrocellulose membranes (Millipore, Bedford, MA, USA). Five percent skim milk was used to block the membranes for 2 h. The membranes were subsequently incubated with the following primary antibodies: monoclonal mouse Anti-iNOS (1:1000; BD Biosciences Pharmingen, CA, USA); polyclonal rabbit anti-mouse COX-2 (1:800; Abcam, Cambridge, MA); polyclonal rabbit anti-mouse SOCS3 (1:1000 Abcam, Cambridge, MA); mouse anti-GAPDH (1:1000; Chemicon, Temecula, CA) and monoclonal anti-α-tubulin (1:1000; Sigma, St. Louis, USA). The membranes were then incubated with the corresponding secondary antibodies (1:10000; Sigma, St. Louis, USA). The protein bands were imaged with a ChemiScope 3300 Mini (CLINX, Shanghai, China). The protein expression levels were normalized to either α-tubulin or GAPDH, which served as the loading controls.

### Statistical analyses

The values are presented as the mean ± SEM. One-way ANOVAs followed by Bonferroni tests were utilized for multiple-group comparisons. Student’s *t*-test was used for comparisons between two groups. A *P*-value of <0.05 was considered statistically significant.

## Results

### Elevated expression of miR-3473b following cerebral ischemia

**Fig. 1:**
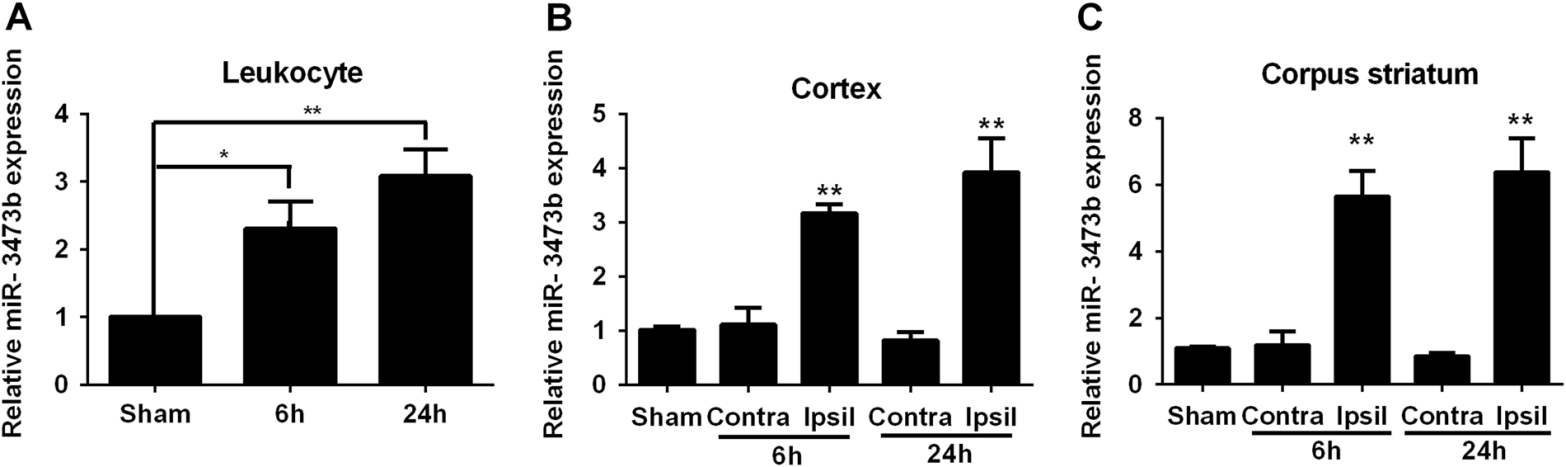
miR-3473b levels increased in mice following MCAO **a** miR-3473b expression in the leukocytes of MCAO mice as detected by RT-PCR (*n* = 5 per group). **b** miR-3473b levels in the cortex of the sham-operated mice, as well as in the ipsilateral and contralateral cortices of the mice receiving 1 h of MCAO followed by 6 h or 24 h of reperfusion (*n* = 6 per group). **c** miR-3473b levels in the striatum of the sham-operated mice, as well as in the ipsilateral and contralateral striatum of the mice receiving 1 h of MCAO followed by 6 h or 24 h of reperfusion (*n* = 6 per group). *P < 0.05 and **P < 0.01, compared with the sham mice or the contralateral hemispheres of the MCAO mice

We used qRT-PCR to assess the expression levels of miR-3473b in mouse leukocytes and brain tissues following MCAO. Compared to the sham-operated mice, mice receiving MCAO displayed a significant increase in levels of miR-3473b in the leukocytes at 6 and 24 h after reperfusion (Fig. 1a). Furthermore, relative to the contralateral side or to the sham-operated brain, miR-3473b levels were also remarkably enhanced in the ipsilateral cortex and striatum after MCAO (Fig. 1b, c).

### Pretreatment with the miR-3473b antagomir reduces infarct damage following MCAO in mice

**Fig. 2:**
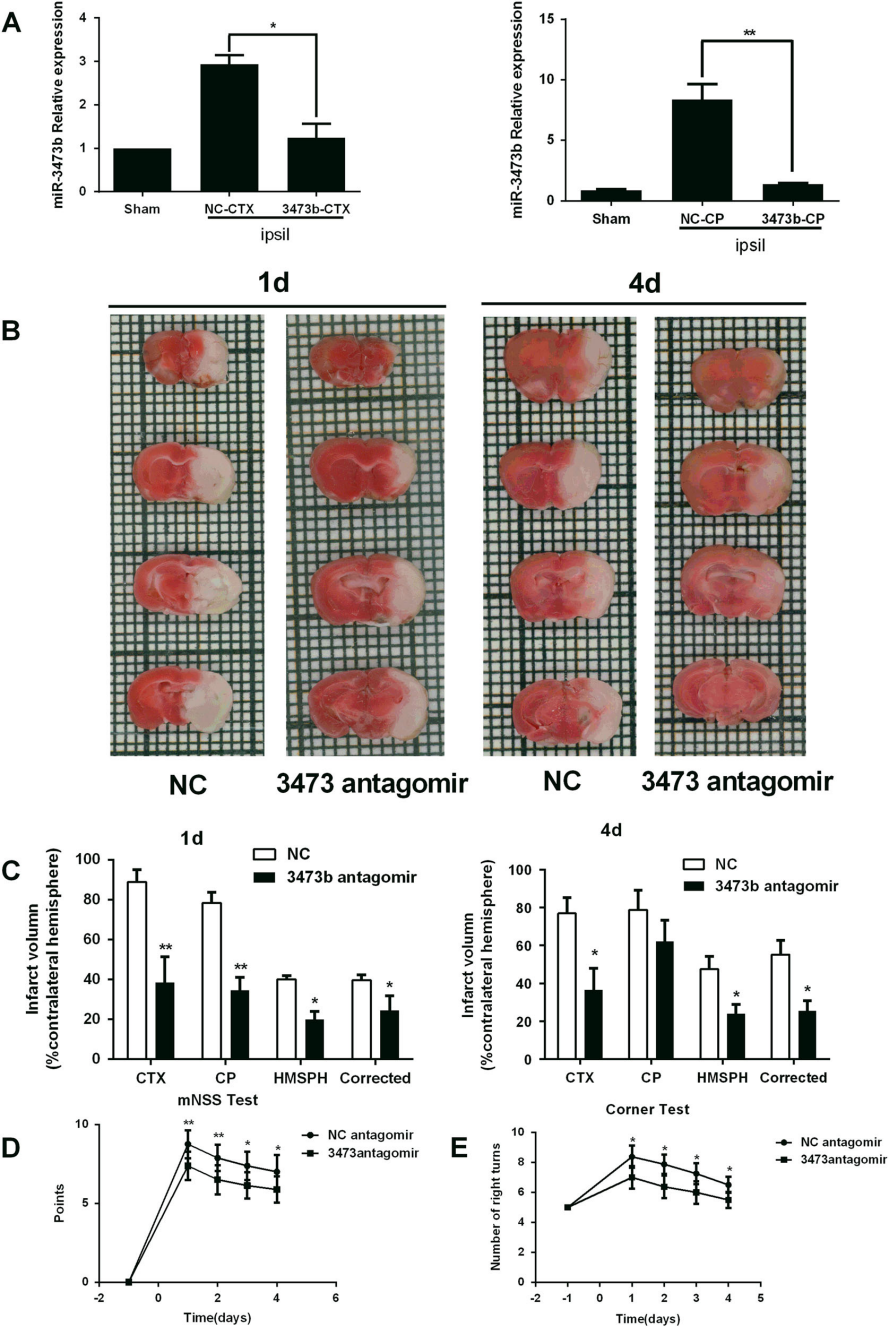
Intracerebroventricular injection of the miR-3473b antagomir reduced acute infarct damage in mice following MCAO **a** miR-3473b levels in the ipsilateral cortex and striatum at 24 h after reperfusion in mice receiving ICV injection of either the miR-3473b or NC antagomirs (*n* = 4 per group). Sham: sham-operated mice without ICV injection. **b** Representative images of TTC staining in brain sections collected from mice receiving ICV injection of either the miR-3473b or NC antagomir at 1 or 4 days after reperfusion. **c** Quantitative data regarding the effects of the miR-3473b antagomir on cerebral infarction as assessed by TTC histology at 1 day (*n* = 8 per group) or 4 days after reperfusion (*n* = 8 per group). **d** The modified neurological severity scores (mNSS) were significantly lower in the miR-3473b antagomir group than the NC antagomir group at 1-4 days after MCAO (*n* = 8 per group). **e** Compared to NC antagomir, miR-3473b antagomir improved the sensorimotor asymmetric abnormalities in the corner test (*n* = 8 per group). NC: mice receiving ICV injection of the NC antagomir; miR-3473b antagomir: mice receiving ICV injection of the miR-3473b antagomir. *CTX* cortex, *CP* striatum, *HMSPH* hemisphere; Corrected: hemisphere infarction corrected for edema. *P < 0.05, **P < 0.01; compared with NC

Next, we investigated the effects of miR-3473b on acute infarct damage. The mice received an intracerebroventricular (ICV) infusion of either the miR-3473b antagomir or control antagomir (NC) 3 days prior to MCAO. Compared to the NC antagomir, the qPCR measurement confirmed that the miR-3473b antagomir attenuated post-ischemic induction of miR-3473b in the ipsilateral cortex and striatum, 24 h after reperfusion in MCAO mice (Fig. 2a). After 1 or 4 days of reperfusion, the mice receiving the miR-3473b antagomir displayed significantly smaller infarct volumes in the cortex, striatum and ipsilateral hemisphere than the mice that received treatment with the NC antagomir (Fig. 2b, c). Moreover, mNSS scoring and the corner tests were performed to assess neurological deficits during the first 4 days following MCAO. The mice receiving the miR-3473b antagomir displayed lower mNSS scores than those treated with control antagomir (NC) (Fig. 2d). In the corner test, the miR-3473b antagomir group also performed better than the NC group (Fig. 2e).

### miR-3473b antagomir inhibits neuroinflammation following MCAO in vivo

**Fig. 3:**
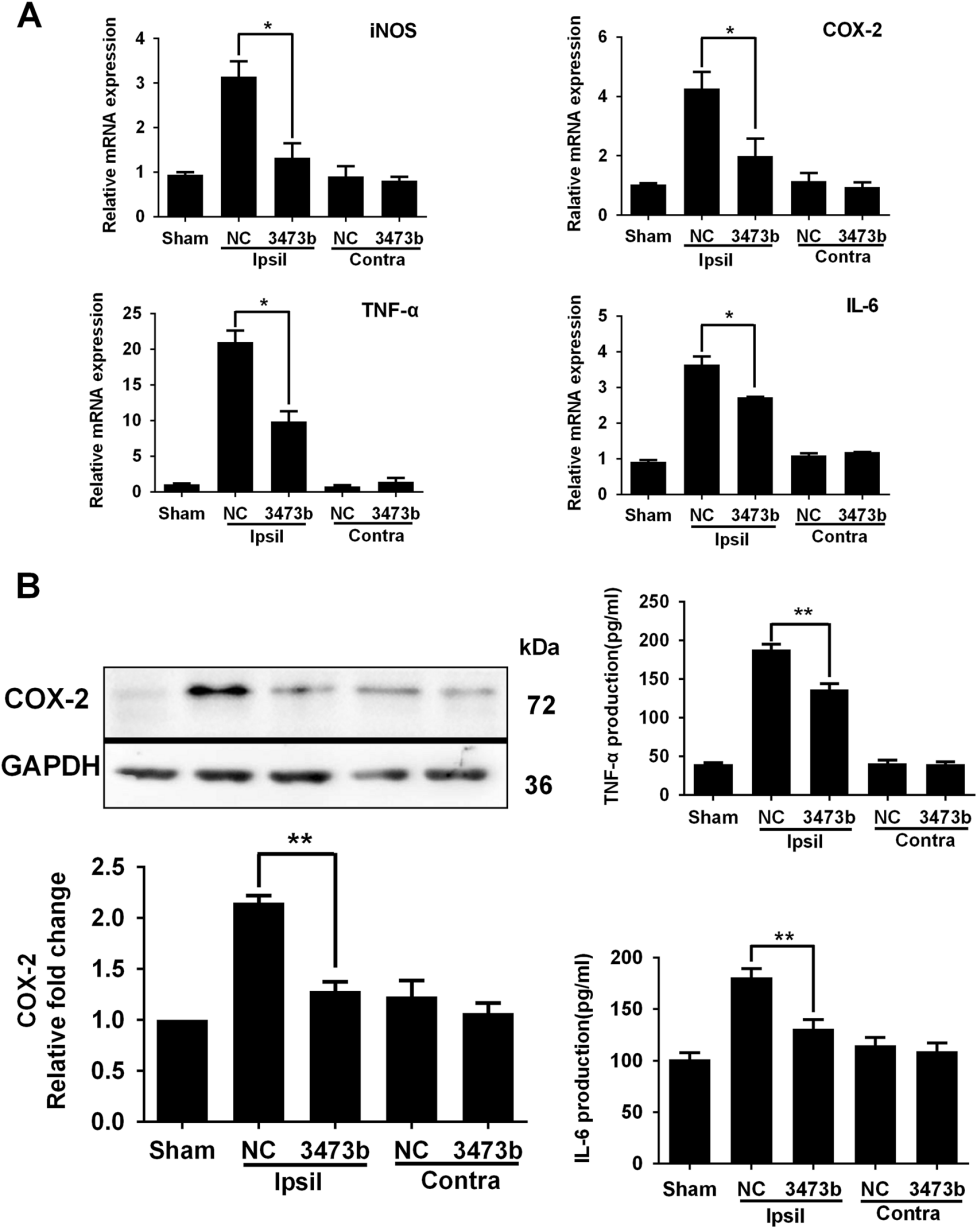
Intracerebroventricular injection of the miR-3473b antagomir inhibited neuroinflammation following MCAO **a** mRNA levels of pro-inflammatory factors (iNOS, COX-2, TNF-α, and IL-6) in the cortical penumbra of mice receiving ICV injection of either the miR-3473b or NC antagomirs (*n* = 4). **b** Western blot and ELISA results showed that the miR-3473b antagomir but not the NC antagomir inhibited post-ischemic induction of the COX-2, IL-6, and TNF-α proteins in the ischemic penumbra (*n* = 3 per group). Sham: Sham operated groups; Contra: contralateral side; Ipsil: ipsilateral side. *P < 0.05, **P < 0.01; compared with NC

We further assessed the effects of miR-3473b on post-ischemic neuroinflammation. Compared to the NC antagomir, the miR-3473b antagomir treatment significantly decreased the mRNA expression of pro-inflammatory factors (iNOS, COX-2, IL-6, and TNF-α) in the mouse cortical penumbra at 24 h after reperfusion (Fig. 3a). Coordinately, the miR-3473b antagomir pretreatment decreased the protein expression of COX-2, TNF-α, and IL-6 in the cortical penumbra (Fig. 3b).

### Enhanced expression of miR-3473b is associated with microglial activation and the inflammatory phenotype

**Fig. 4:**
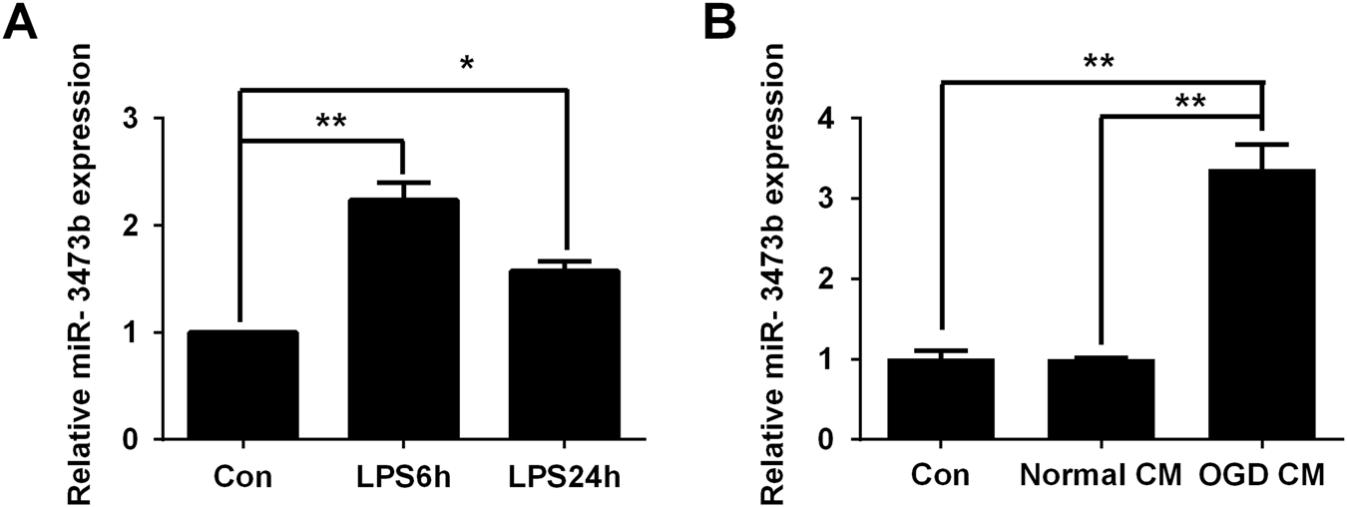
Increased expression of miR-3473b in BV2 microglia stimulated to induce the inflammatory phenotype **a** Compared to the control group treated with vehicle, the BV2 cells stimulated with LPS showed a significant increase in miR-3473b expression at 6 and 24 h after LPS treatment (*n* = 3 per group). **b** BV2 cells treated with conditioned medium collected from OGD neurons also displayed enhanced expression of miR-3473b compared to BV2 cells treated with normal neuronal medium (Normal CM) or control BV2 microglia without treatment (*n* = 4 per group). Data are representative of three to four independent experiments. *P < 0.05, **P < 0.01; compared with the control groups or the Normal CM groups

We used cultured BV2 microglial cells to investigate the effects of miR-3473b on microglia-mediated neuroinflammation in vitro. The BV2 cells were stimulated with lipopolysaccharide (LPS) (200 ng/ml), a well-established activator of microglia/macrophages. Compared to the vehicle, the BV2 microglial cells stimulated with LPS showed a significant induction of miR-3473b at 6 and 24 h (Fig. 4a). To better mimic in vivo cerebral ischemia, we used conditioned medium collected from OGD-treated primary neurons (neuronal OGD CM) to stimulate the BV2 microglia as previously reported. After 24 h of incubation, miR-3473b expression was significantly enhanced in the BV2 microglial cells treated with the OGD neuronal medium compared to the cells treated with normal neuronal medium (Fig. 4b).

### miR-3473b antagomir suppresses microglia-mediated neuroinflammation

**Fig. 5:**
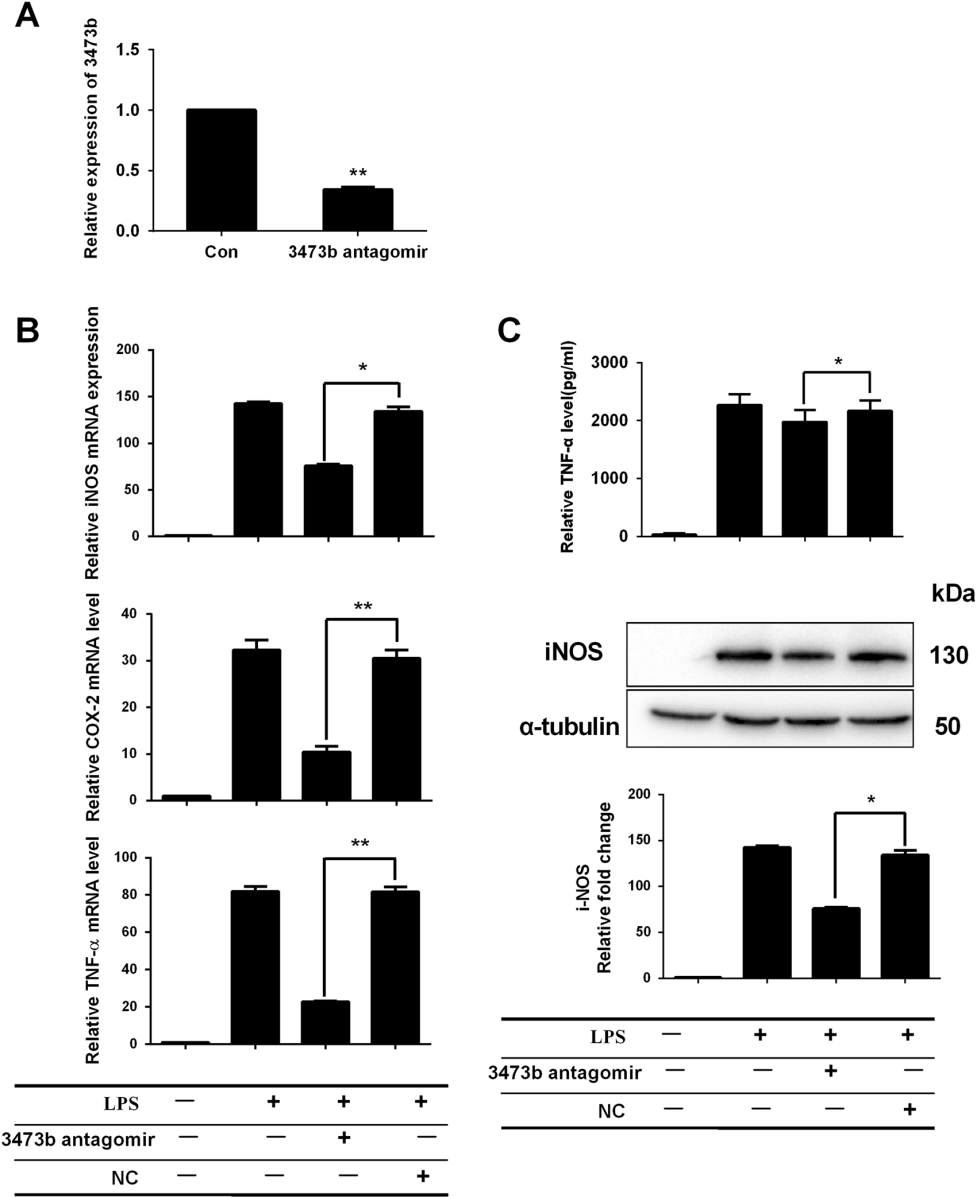
miR-3473b antagomir suppressed the expression of pro-inflammatory mediators in BV2 microglia stimulated with LPS **a** The endogenous expression of miR-3473b decreased in BV2 cells transfected with the miR-3473b antagomir vs. the NC antagomir. **b** The miR-3473b antagomir, but not the NC antagomirs, inhibited the expression of iNOS, COX-2, and TNF-α mRNA in LPS-treated BV2 microglia. **c** The miR-3473b antagomir, but not the NC antagomir, inhibited expression of iNOS and TNF-α protein in LPS-treated BV2 microglia. Data are representative of three independent experiments. *P < 0.05 and **P < 0.01 compared between the indicated groups

**Fig. 6:**
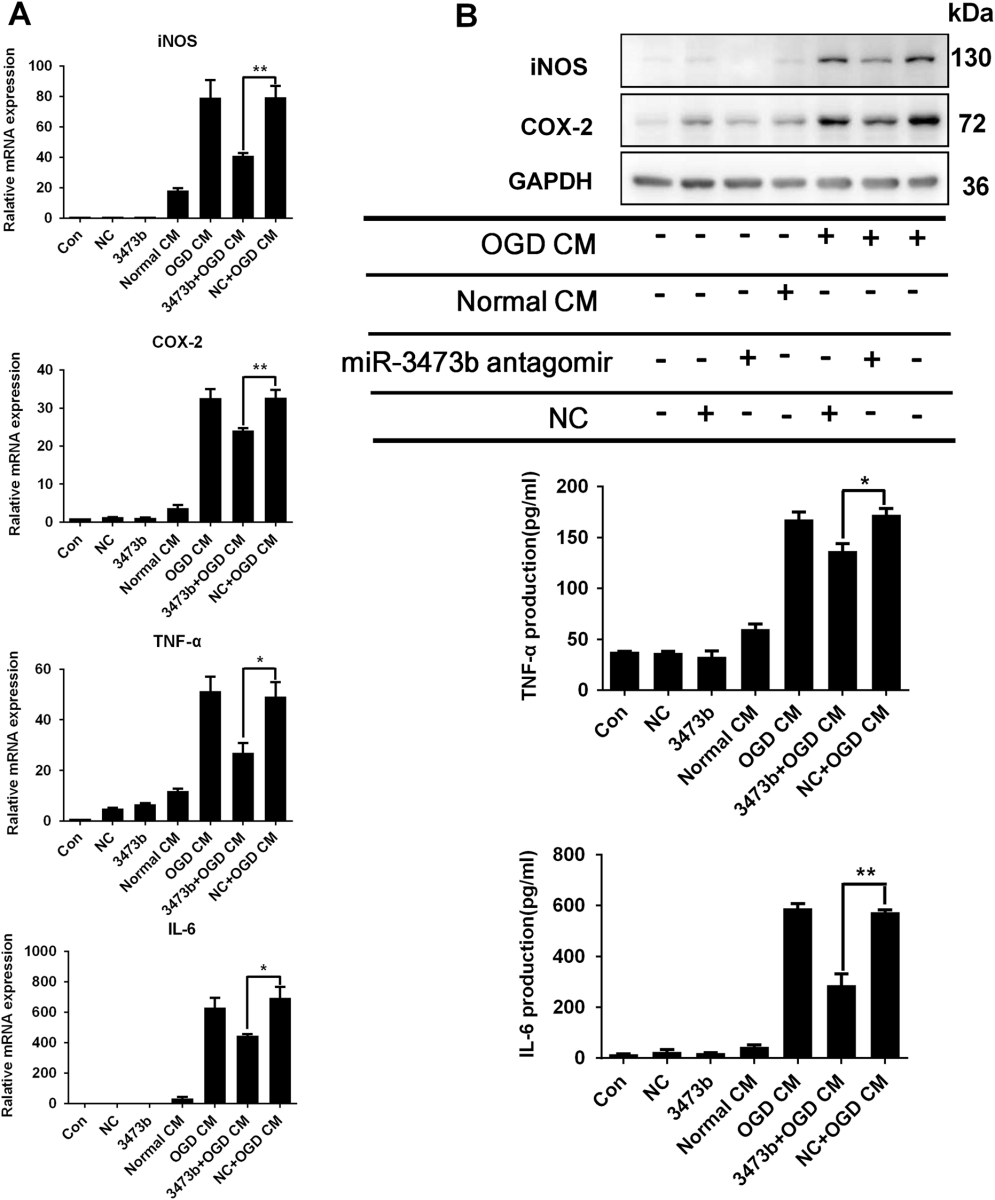
miR-3473b antagomir suppressed the expression of the pro-inflammatory mediators in BV2 microglia stimulated with conditioned medium collected from neurons subjected to OGD (OGD CM) **a** The miR-3473b antagomir, but not the NC antagomir, attenuated OGD CM-induced expression of iNOS, COX-2, TNF-α, and IL-6 mRNA in BV2 microglia (*n* = 4 for iNOS, COX-2; *n* = 3 for TNF-α, IL-6). **b** The miR-3473b antagomir, but not the NC antagomir, attenuated OGD CM-induced expression of the iNOS, COX-2, TNF-α and IL-6 proteins in BV2 microglia. Data are representative of three to four independent experiments. *P < 0.05 and **P < 0.01 compared between the indicated groups

To further elucidate whether the enhanced expression of miR-3473b contributed to microglia-mediated neuroinflammation, we transfected BV2 cells with miR-3473b antagomirs to reduce the endogenous expression of miR-3473b in the BV2 microglia (Fig. 5a). At 48 h after transfection, the BV2 cells were activated with LPS. Compared to the NC antagomir control, the miR-3473b antagomir pretreatment significantly attenuated the LPS-induced mRNA (iNOS, COX-2 and TNF-α) (Fig. 5b) and protein expression of the pro-inflammatory mediators (iNOS and TNF-α) (Fig. 5c). We further investigated whether the miR-3473b antagomir suppressed neuroinflammation in BV2 cells treated with neuronal OGD CM. At 48 h after transfection with either the miR-3473b or NC antagomir, neuronal OGD CM-induced mRNA and protein expression of the pro-inflammatory mediators (iNOS, COX-2, TNF-α, and IL-6) in BV2 microglia were significantly attenuated by miR-3473b antagomir treatment (Fig. 6).

### SOCS3 is a potential target of miR-3473b

**Fig. 7:**
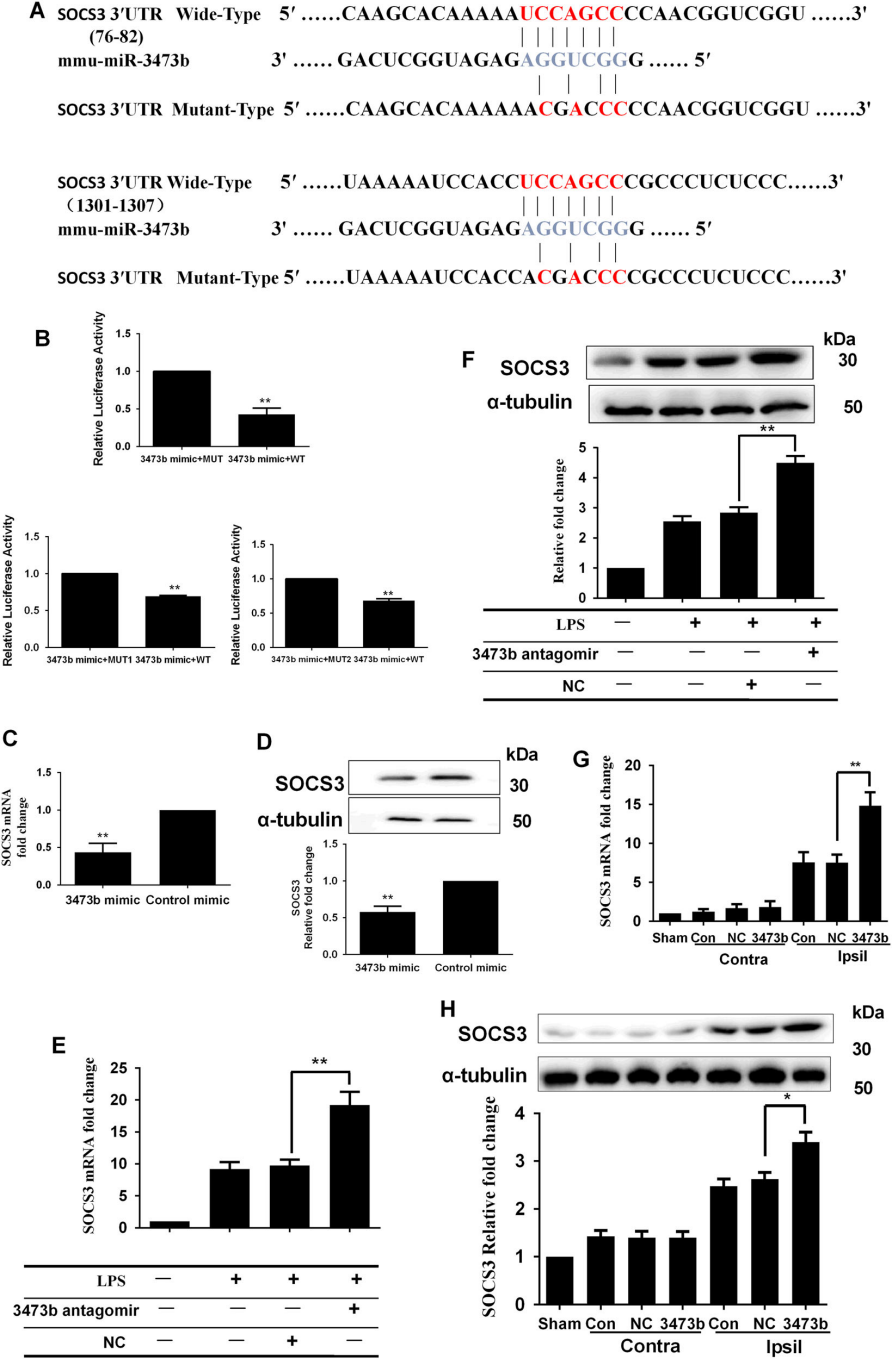
SOCS3 3′-untranslated region (UTR) was directly targeted by miR-3473b **a** Schema of the WT and mutated SOCS3 3′-UTR indicating the interaction sites between miR-3473b and the 3′-UTR of SOCS3. **b** Dual luciferase assay in HEK293 cells co-transfected with the miR-3473b mimic and reporter vectors containing either the wild-type or mutated 3’-UTR of SOCS3. The Renilla luciferase activity was normalized to the Firefly luciferase activity (*n* = 4–5 per group). MUT: the construct with the mutated nucleotides in both binding sites; MUT1: the construct with the mutated nucleotides in the first binding site between 76–82; MUT2: the construct with the mutated nucleotides in the second binding site between 1301–1307. **c** The miR-3473b mimic, but not the NC mimic, inhibited the mRNA expression of SOCS3 in the BV2 microglia (*n* = 6 per group). **d** The miR-3473b mimic, but not the NC mimic, inhibited the protein expression of SOCS3 in BV2 microglia (*n* = 3 per group). **e** miR-3473b antagomir increased the SOCS3 mRNA expression in the LPS-treated BV2 cells (*n* = 5 per group). **f** miR-3473b antagomir increased the SOCS3 protein expression in the LPS-treated BV2 cells (*n* = 4 per group). **g** mRNA levels of SOCS3 in the cortical tissue of sham-operated and MCAO mice treated with miR-3473b or NC antagomir (*n* = 5 per group). **h** Protein levels of SOCS3 in the cortical tissue of sham-operated and MCAO mice treated with miR-3473b or NC antagomirs (*n* = 4 per group). Contra: contralateral side; Ipsil: ipsilateral side. *P < 0.05 and **P < 0.01 compared between the indicated groups

To explore the signaling pathway of miR-3473b-modulated neuroinflammation, we identified the potential gene targets of miR-3473b by using the miRDB program (http://mirdb.org/miRDB/). Among all of the predicted gene targets, SOCS3 was chosen as a candidate because it is reported to be involved in inflammation. Two potential binding sites in the 3′-UTR of the SOCS3 mRNA were identified as being targeted by miR-3473b (Fig. 7a). To obtain direct evidence that SOCS3 was a target of miR-3473b, the fragment of the SOCS3 3′-UTR containing the nucleotides complementary to miR-3473b (nucleotides 76–82 and 1301–1307 of the SOCS3 3′-UTR) was cloned into a luciferase reporter plasmid (pmiRGLO), such that the SOCS3 3′-UTR was placed downstream of the luciferase reporter gene. Furthermore, we constructed three plasmids containing the mutated nucleotides in the first binding site (nucleotides 76–82), the second binding site (nucleotides 1301–1307) or both the above sites of the SOCS3 3′-UTR. Plasmids containing either the wild-type or mutant SOCS3 3′-UTR were then co-transfected with the miR-3473b mimic in HEK 293 cells. The miR-3473b mimic inhibited luciferase activity in the HEK 293 cells transfected with the wild-type SOCS3 3′-UTR, but not in the cells transfected with the mutated SOCS3 3′-UTR (Fig. 7b). The data suggested both binding sites of 3’-UTR of SOCS3 are targeted by miR-3473b. Moreover, the miR-3473b mimic, but not the control mimic (NC), reduced endogenous mRNA and protein expression of SOCS3 in BV2 microglia (Fig. 7c, d). Finally, we examined whether miR-3473b antagomir inhibited microglial activation by targeting SOCS3 in the BV2 cells treated with LPS and in the ischemic brain. As shown in Figs. 7e, f, compared to the NC group, the miR-3473b antagomir significantly increased both SOCS3 mRNA and protein levels in LPS-stimulated BV2 cells. To further define the functional relationship between miR-3473b and SOCS3 after MCAO, we assessed mRNA and protein levels of SOCS3 in mice treated with miR-3473b antagomir or NC antagomir following MCAO. Compared to the NC antagomir, the miR-3473b antagomir treatment significantly increased the mRNA expression of SOCS3 in the mouse cortical penumbra at 24 h after reperfusion (Fig. 7g). Consistently, the miR-3473b antagomir pretreatment increased the protein expression of SOCS3 in the cortical penumbra of MCAO mice (Fig. 7h).

These results suggested that miR-3473b likely binds to the 3′-UTR of SOCS3 and reduces SOCS3 expression in microglia.

## Discussion

In the present study, we investigated the effects of miR-3473b on cerebral ischemia injury. By using a well-established mouse transient MCAO model, we showed that miR-3473b expression was significantly increased in the ischemic brain. Inhibiting post-ischemic cerebral induction of miR-3473b by pretreating with a miR-3473b antagomir reduced acute tissue damage and neurologic deficits following MCAO. When either LPS or conditioned medium collected from cultured OGD neurons were used to stimulate microglial cells to induce the pro-inflammatory phenotype in vitro, miR-3473b expression was also enhanced. Moreover, downregulation of miR-3473b by its antagomir reduced microglia-mediated neuroinflammation. Lastly, we showed that SOCS3 was directly targeted by miR-3473b. The results revealed, at first time, that miR-3473b plays an important role in microglia-mediated inflammation, and alterations in miR-3473b expression contribute to stroke pathogenesis.

miRNAs play an essential role under various pathophysiological conditions. Several previous studies have reported that miRNAs are mediators of ischemic injury^10,18^. Microglia are the primary immune cells in the brain. Microglial activation is considered to be a hallmark of neuroinflammation, which has been shown to contribute to the pathogenesis associated with multiple brain disorders, including ischemic injuries and neurodegenerative diseases. Significant effort has been devoted to investigating the role of microglia in post-stroke neuroinflammation and pathology^19–22^. However, the exact role of microglial cells following stroke still remains elusive^1,19,23^. It is generally believed that cerebral ischemia triggers microglial activation. The activated microglia then produce detrimental pro-inflammatory cytokines (iNOS, COX-2, TNF-α, and IL-6^24^), which contribute to secondary brain ischemic injury and represent the main cause of cerebral injury aggravation^25,26^. The present data demonstrate that the activation of microglia by LPS or conditioned medium collected from cultured OGD neurons increased the expression of miR-3473b. Furthermore, the elevated expression of miR-3473b is associated with enhanced production of pro-inflammatory factors in BV2 cells. The downregulation of miR-3473b by its antagomir attenuated the microglial activation in both the cultured BV2 cells and the ischemic brain following MCAO in animals. The present data elucidate the functional role of miR-3473b. The elevation of miR-3473b levels in the ischemic brain and its association with inflammation not only indicates that the miRNA is involved in the pathophysiological development of stroke, but may also reveal a potential novel strategy for stroke treatment.

MiRNAs target partially complementary 3′-UTR sequences of mRNAs, leading to the downregulation of protein expression^27^. Computational analysis identified SOCS3, a member of the STAT-induced STAT inhibitor family, as a potential target of miR-3473b. The 3′-UTR of the SOCS3 mRNA contained two miR-3473b binding sites. We demonstrated that miR-3473b regulates SOCS3 expression by targeting these 3′-UTR sites, as evidenced by the results from the dual luciferase assay. Moreover, the miR-3473b mimic decreased endogenous expression of SOCS3 in BV2 microglia. More relevantly, the miR-3473b antagomir significantly increased the expression of SOCS3 in both mRNA and protein levels in LPS-stimulated BV2 cells. The data from in vivo studies also showed that the miR-3473b antagomir pretreatment increased the mRNA and protein expression of SOCS3 in the cortical penumbra of the MCAO mice. Moreover, miR-3473b antagomir inhibited LPS-mediated inflammation in BV2 cells, suppressed neuroinflammation and decreased acute infarct damage in mice following MCAO.

These results suggested that the enhanced expression of SOCS3 by the miR-3473b antagomir may in part contribute to the beneficial effects of this molecular on MCAO-induced acute infarct damage and neuroinflammation.

However, it has been reported that LPS alone also was shown to stimulate SOCS3 mRNA and protein levels in vitro^28^. We observed here that both SOCS3 mRNA and protein levels were increased in the ipsilateral cortices from MCAO mice as compared to the sham-operated mice. These results were consistent with the previous report showing the upregulation of SOCS3 mRNA expression in the hippocampal CA1 region following transient forebrain ischemia^29^. Thus, the upregulation of SOCS3 under the LPS stimulation or ischemia conditions is unlikely to be associated with miR-3473b; other mechanism may underlie this effect.

SOCS3 is an intracellular, cytokine-inducible protein that inhibits cytokine signaling in numerous cell types, including cells found in the immune and central nervous systems^30^. The SOCS family is composed of eight members: cytokine-inducible SRC homology 2 (SH2)-domain-containing protein (CIS) and SOCS 1-7^31,32^. SOCS3 exerts a broad effect on the immune response by inhibiting the signaling molecules of the immune system^31,33^, particularly the IL-6 family of cytokines^18^. SOCS3 expression has been shown to display protective effects against inflammatory diseases^31,34^. Thus, it is likely that miR-3473b acts through microglial SOCS3 to modulate post-ischemic neuroinflammation and consequently protect neurons from ischemic injury. Although the miR-3473b knockdown studies suggest that miR-3473b may play a role through regulation of SOCS expression, we cannot exclude other possible targets or mechanisms. Further blockade or supplement of SOCS3 studies are needed to demonstrate the role of SOCS in miR-3473b contribution to stroke pathology.

In addition, it is interesting to note that we detected a significant increase of miR-3473b in the leukocytes of MCAO mice. However, according to the current version of microRNA database miRBase, miR-3473b homolog has not been identified in human. Therefore, whether or not miR-3473b can be used as a diagnostic biomarker or therapeutic target for stroke recovery requires further investigation.

In summary, our study showed that post-ischemic induction of miR-3473b contributed to cerebral ischemic injury through the enhancement of microglia-mediated neuroinflammation by targeting SOCS3. Inhibiting post-ischemic induction of miR-3473b may represent a potential therapeutic target for ischemic stroke.

## References

[1] Ma Y, Wang J, Wang Y, Wang G Y (2017). The biphasic function of microglia in ischemic stroke. Prog. Neurobiol.

[2] Kim JY, Kim N, Yenari MA (2015). Mechanisms and potential therapeutic applications of microglial activation after brain injury. CNS. Neurosci. Ther..

[3] Moskowitz MA, Lo EH, Iadecola C (2010). The science of stroke: mechanisms in search of treatments. Neuron..

[4] Jin Q (2014). Improvement of functional recovery by chronic metformin treatment is associated with enhanced alternative activation of microglia/macrophages and increased angiogenesis and neurogenesis following experimental stroke. Brain. Behav. Immun..

[5] Chan PH (2001). Reactive oxygen radicals in signaling and damage in the ischemic brain. J. Cereb. Blood Flow Metab..

[6] Rink C, Khanna S (2011). MicroRNA in ischemic stroke etiology and pathology. Physiol. Genom..

[7] Hamzei Taj S, Kho W, Riou A, Wiedermann D, Hoehn M (2016). MiRNA-124 induces neuroprotection and functional improvement after focal cerebral ischemia. Biomaterials..

[8] Ouyang YB, Stary CM, Yang GY, Giffard R (2013). microRNAs: innovative targets for cerebral ischemia and stroke. Curr. Drug. Target..

[9] Saugstad JA (2010). MicroRNAs as effectors of brain function with roles in ischemia and injury, neuroprotection, and neurodegeneration. J. Cereb. Blood Flow Metab.

[10] Ni J (2015). MicroRNA let-7c-5p protects against cerebral ischemia injury via mechanisms involving the inhibition of microglia activation. Brain. Behav. Immun..

[11] Wu C (2014). IFN-gamma primes macrophage activation by increasing phosphatase and tensin homolog via downregulation of miR-3473b. J. Immunol..

[12] Tao Z (2015). Neuroprotective effect of microRNA-99a against focal cerebral ischemia-reperfusion injury in mice. J. Neurol. Sci..

[13] Liu L (2016). Calcium/calmodulin-dependent protein kinase kinase beta is neuroprotective in stroke in aged mice. Eur. J. Neurosci..

[14] Doeppner T. R., Kaltwasser B., Bahr M., Hermann D. M. Effects of neural progenitor cells on post-stroke neurological impairment-a detailed and comprehensive analysis of behavioral tests. (1662-5102 (Linking)). 10.3389/fncel.2014.00338 PMC4205824 25374509

[15] Gan P (2015). Anti-inflammatory effects of glaucocalyxin B in microglia cells. J. Pharmacol. Sci..

[16] Tan MS (2014). IL12/23 p40 inhibition ameliorates Alzheimer’s disease-associated neuropathology and spatial memory in SAMP8 mice. J. Alzheimer’s Dis.

[17] Hu X (2012). Microglia/macrophage polarization dynamics reveal novel mechanism of injury expansion after focal cerebral ischemia. Stroke..

[18] Xu Z (2015). Design, synthesis and evaluation of a series of non-steroidal anti-inflammatory drug conjugates as novel neuroinflammatory inhibitors. Int. Immunopharmacol..

[19] Qin X (2012). Toll-like receptor 4 signaling is involved in PACAP-induced neuroprotection in BV2 microglial cells under OGD/reoxygenation. Neurol. Res..

[20] Zhao H (2013). MiRNA-424 protects against permanent focal cerebral ischemia injury in mice involving suppressing microglia activation. Stroke..

[21] Zheng S (2015). Sphingosine kinase 1 mediates neuroinflammation following cerebral ischemia. Exp. Neurol..

[22] Karthikeyan A, Patnala R, Jadhav SP, Ling EA, Dheen ST (2016). MicroRNAs: Key players in microglia and astrocyte mediated inflammation in CNS pathologies. Curr. Med. Chem..

[23] Liu P (2015). MicroRNA-424 protects against focal cerebral ischemia and reperfusion injury in mice by suppressing oxidative stress. Stroke..

[24] Zhao YY (2011). TSPO-specific ligand vinpocetine exerts a neuroprotective effect by suppressing microglial inflammation. Neuron. Glia. Biol..

[25] Huang J, Upadhyay UM, Tamargo RJ (2006). Inflammation in stroke and focal cerebral ischemia. Surg. Neurol..

[26] Caballero-Garrido E (2015). In Vivo Inhibition of miR-155 Promotes Recovery after Experimental Mouse Stroke. J. Neurosci.

[27] Stary CM (2015). MicroRNA-200c contributes to injury from transient focal cerebral ischemia by targeting Reelin. Stroke..

[28] Qin H (2007). Molecular mechanism of lipopolysaccharide-induced SOCS-3 gene expression in macrophages and microglia. J. Immunol..

[29] Choi JS (2008). Induction of suppressor of cytokine signaling-3 in astrocytes of the rat hippocampus following transient forebrain ischemia. Neurosci. Lett..

[30] Collins AS, McCoy CE, Lloyd AT, O’Farrelly C, Stevenson NJ (2013). miR-19a: an effective regulator of SOCS3 and enhancer of JAK-STAT signalling. PLoS. ONE..

[31] Baker BJ, Akhtar LN, Benveniste EN (2009). SOCS1 and SOCS3 in the control of CNS immunity. Trend. Immunol..

[32] Yin Y, Liu W, Dai Y (2015). SOCS3 and its role in associated diseases. Hum. Immunol..

[33] Zhang B (2016). Mer receptor tyrosine kinase negatively regulates lipoteichoic acid-induced inflammatory response via PI3K/Akt and SOCS3. Mol. Immunol..

[34] Qin H (2016). Correction: SOCS3 Deficiency Promotes M1 Macrophage Polarization and Inflammation. J. Immunol..

